# Supplementary material for: Maternal Plasma Glycerophospholipids LC-PUFA Levels Have a Sex-Specific Association with the Offspring’s Cord Plasma Glycerophospholipids-Fatty Acid Desaturation Indices at Birth
Source: Int J Environ Res Public Health. 2022 Nov 11;19(22):14850. doi: 10.3390/ijerph192214850 (PMC9691092; doi:10.3390/ijerph192214850)
Supplement: Supplementary file 1 [file ijerph-19-14850-s001.zip › ijerph-1894987-supplementary.pdf]

Table S1. Nutritional questionnaire results

Intakes/day	Mothers of male offspring	Mothers of female offspring
	(n=73)	(n=80)
**Energy (kcal/day)**	1988 ± 490	1995 ± 550
**Carbohydrates (%)**	62 ± 8.1	64 ± 5.7
**Proteins (%)**	12 ± 1.9	12 ± 1.5
**Lipids (%)**	26 ± 5.1	24 ± 8.1
**Saturated Fatty acids (%)**	9.4 ± 2.9	9.8 ± 5.9
**Monounsaturated fatty acids (%)**	6.4 ± 2.1	7.1 ± 3.4
**Linoleic acid (%), n-6**	9.1 ± 4.1	9.8 ± 3.1
**Alpha-Linolenic acid (%), n3**	0.2 ± 0.05	0.1 ± 0.02
**Gamma-Linolenic acid (%), n6**	0.01 ± 0.05	0.01 ± 0.05
**Eicosapentaenoic acid (g), n3**	0.01 ± 0.001	0.01 ± 0.001
**Docosahexaenoic acid (g), n3**	0.02 ± 0.001	0.018 ± 0.01

Data are expressed as mean ± SD, for carbohydrates, proteins, and lipids percentage of total caloric intake as indicated

Table S2. Correlation between plasma GP-FA% of mothers and cord blood of their male new-borns

FA (%)	r	CI 95%	P-value
**SFA**			
C14	0.53	0.34 to 0.68	<0.0001
C16	0.33	0.11 to 0.52	0.0044
C18	0.07	-0.16 to 0.29	0.544
**MUFA**			
C16:1n7	0.19	-0.04 to 0.41	0.101
C18:1n9	0.25	0.02 to 0.46	0.033
**n-6 PUFA**			
C18:2n6 (LA)	0.32	0.09 to 0.51	0.007
C20:3n6 (DHGLA)	0.44	0.23 to 0.60	0.0001
C20:4n6 (ARA)	0.24	0.008 to 0.44	0.043
**n-3 PUFA**			
C20:5 (EPA)	0.11	-0.12 to 0.33	0.351
C22:5 (DPA)	0.16	-0.07 to 0.3745	0.182
C22:6 (DHA)	0.34	0.12 to 0.53	0.004
**∑SFA**	0.09	-0.15 to 0.32	0.457
**∑MUFA**	0.25	0.025 to 0.46	0.031
**∑PUFA**	0.24	0.004 to 0.45	0.04
**∑n-6**	0.23	-0.01 to 0.44	0.046
**∑n-3**	0.42	0.21 to 0.59	0.0003
**∑n-6/n-3**	0.36	0.14 to 0.55	0.002

Table S3. Relationship between plasma GP-FA% of mothers and cord blood of their female new-borns

FA (%)	r	CI 95%	P-value
**SFA**			
C14	0.363	0.16 to 0.54	0.001
C16	0.225	0.004 to 0.425	0.04
C18	0.333	0.122 to 0.515	0.003
**MUFA**			
C16:1n7	0.359	0.149 to 0.539	0.0012
C18:1n9	0.444	0.245 to 0.605	<0.0001
**PUFA n-6**			
C18:2n6 (LA)	0.349	0.140 to 0.529	0.0015
C20:3n6 (DHGLA)	0.319	0.106 to 0.505	0.004
C20:4n6 (ARA)	0.302	0.08 to 0.492	0.007
**PUFA n-3**			
C20:5 (EPA)	0.137	-0.086 to 0.346	0.226
C22:5 (DPA)	0.356	0.144 to 0.536	0.0014
C22:6 (DHA)	0.592	0.428 to 0.718	<0.0001
∑**SFA**	0.416	0.216 to 0.582	0.0001
∑**MUFA**	0.393	0.191 to 0.565	0.0003
∑**PUFA**	0.338	0.126 to 0.519	0.0022
∑**n-6**	0.259	0.042 to 0.453	0.02
∑**n-3**	0.487	0.2998 to 0.6387	<0.0001
∑**n-6/n-3**	0.417	0.217 to 0.583	0.0001
